# Isoflavones Reduce Copper with Minimal Impact on Iron *In Vitro*

**DOI:** 10.1155/2015/437381

**Published:** 2015-07-26

**Authors:** Jana Karlíčková, Kateřina Macáková, Michal Říha, Liliane Maria Teixeira Pinheiro, Tomáš Filipský, Veronika Horňasová, Radomír Hrdina, Přemysl Mladěnka

**Affiliations:** ^1^Department of Pharmaceutical Botany and Ecology, Faculty of Pharmacy in Hradec Králové, Charles University in Prague, Heyrovského 1203, 500 05 Hradec Králové, Czech Republic; ^2^Department of Pharmacology and Toxicology, Faculty of Pharmacy in Hradec Králové, Charles University in Prague, Heyrovského 1203, 500 05 Hradec Králové, Czech Republic; ^3^Faculty of Pharmacy, University of Porto, Praça Gomes Teixeira, 4099-002 Porto, Portugal

## Abstract

Isoflavones are commonly consumed in many Asian countries and have potentially positive effects on human being. Only a few and rather controversial data on their interactions with copper and iron are available to date. 13 structurally related isoflavones were tested in the competitive manner for their Cu/Fe-chelating/reducing properties. Notwithstanding the 5-hydroxy-4-keto chelation site was associated with ferric, ferrous, and cupric chelation, the chelation potential of isoflavones was low and no cuprous chelation was observed. None of isoflavones was able to substantially reduce ferric ions, but the vast majority reduced cupric ions. The most important feature for cupric reduction was the presence of an unsubstituted 4′-hydroxyl; contrarily the presence of a free 5-hydroxyl decreased or abolished the reduction due to chelation of cupric ions. The results from this study may enable additional experiments which might clarify the effects of isoflavones on human being and/or mechanisms of copper absorption.

## 1. Introduction

Isoflavonoids form a subclass of flavonoids which is characterised by aromatic ring B attached at position C_3_ in contrast to the majority of flavonoids which have this ring at C_2_. Isoflavones, which occur in the form of aglycones or as glycosides, are the most common compounds of this class. Although they were thought to be specific only for plants from family Fabaceae, they have been identified in number of gymnosperms, dicots, and monocots and also in some products made from them (beer and bourbon) until now [1–3].

Isoflavones are specific in comparison to other flavonoids due to their slight estrogenic effects [4]. Moreover, they appear to have some positive cardiovascular effect similar to other flavonoids, at least according to a few epidemiological studies [5–7]. However, not all of the epidemiological studies have confirmed an inverse relationship between higher consumption of isoflavonoids and cardiovascular or total mortality [8, 9]. One of the factors may be the dual role of isoflavones, which can act both as antioxidants and as prooxidants depending on their concentrations and conditions [10–13]. Transition metals, copper and iron, represent apparently one of the most important factors influencing the possible pro- or antioxidant activity of isoflavones. Likely due to several methodological problems and controversy, data on iron/copper-chelating properties of isoflavones have been rather limited [10, 13–15]; the relationships between the structure and iron/copper chelation/reduction activity have been still missing. The type of interaction of isoflavonoids with copper might have different clinical outcomes depending on (patho)physiological state of the organism: (1) in the gastrointestinal tract, isoflavones might influence copper and iron absorption; (2) when absorbed, they could chelate transition metals and increase their excretion or they could reduce them and potentially increase the production of the hydroxyl radical, the most reactive known biological reactive oxygen species. Since the bioavailability of common isoflavones is quite low in general [16], probably their effects in the gastrointestinal tract might be of a higher clinical importance.

An interesting topic is the copper absorption in the gastrointestinal tract. Despite a series of papers, there are a lot of unanswered questions concerning copper absorption and the regulation of this process. In particular, is copper absorbed in its reduced cuprous (Cu^+^) or oxidized cupric (Cu^2+^) form or both? Is the transport system common for copper and iron?

The current knowledge attributes the key role in copper oral absorption to the human copper transporter (hCTR1) [17], a high-affinity copper transporter which forms a channel transporting copper in its reduced cuprous form [18]. Although this transporter is essential for bioavailability of copper [17], there are data showing its insignificance for copper absorption from the gastrointestinal lumen supported by a large discussion about its intracellular localization as well [17, 19–21]. On the other side, the role of other possible transporters in copper absorption is ambiguous. Contradictory results can be found concerning DMT1 (DCT1), the ferrous transporter: it has been proposed to be Cu^2+^ transporter [22] and even Cu^+^ transporter [23], while another finding does not support the role of DMT1 in copper transport [24]. Other copper-transporting mechanisms could be as follows: hCTR2 [20], an ATP-dependent high-affinity Cu^2+^ transport [25], and anion transport system which would transport equally Cu^+^ and Cu^2+^ in the chloride-dependent pathway [26]. In principle, a compound having no influence on iron (reduction and chelation) and specific influence on copper (reduction or chelation) could be useful for the further research in this area. Our previous data have supposed that some of such compounds could be found in a group of isoflavones.

Therefore, this study was aimed at detailed* in vitro* analysis of copper and iron interaction with commercially available isoflavones in order to establish structure-activity relationship with identification of the structure(s) for a future copper pharmacokinetic study.

## 2. Materials and Methods

### 2.1. Reagents and Solutions

**Figure 1:**
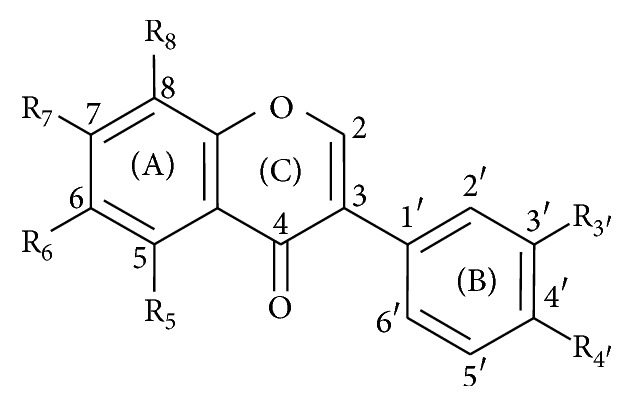
Chemical structure of isoflavones used in this study (see Table 1).

Stock solutions of cupric ions (cupric sulphate pentahydrate, CuSO_4_·5H_2_O), ferric ions (ferric chloride hexahydrate, FeCl_3_·6H_2_O), and ferrous ions (ferrous sulphate heptahydrate, FeSO_4_·7H_2_O) were prepared in water (Milli-Q RG, Merck Millipore, Massachusetts, USA) while that of cuprous ions (cuprous chloride, CuCl) was prepared in the aqueous solution of 0.1 M HCl and 1 M NaCl. The corresponding fresh working solutions (0.25 mM) were prepared by dilution in DMSO (BCS method) or distilled water (hematoxylin and ferrozine method). Hydroxylamine hydrochloride, ferrozine, and bathocuproinedisulfonic acid disodium salt (BCS) were dissolved in distilled water. Hematoxylin was dissolved in DMSO and its working solution (0.25 mM) was usable for no longer than 90 min. All isoflavones (Figure 1 and Table 1) were dissolved in DMSO.

Experiments were performed in 15 mM buffers, acetate (pH 4.5 and 5.5) and HEPES (pH 6.8 and 7.5).

Daidzin, formononetin, genistin, glycitein, glycitin, ononin, prunetin, and puerarin were purchased from Extrasynthese (France); isoformononetin and cladrin were from PhytoLab (Germany). All other chemicals including genistein and daidzein were purchased from Sigma-Aldrich (Germany).

### 2.2. Copper/Iron Chelation and Reduction Assessment

The principle of this method is that oxidized transition metals, cupric or ferric ions, do not react with the indicators (BCS or ferrozine). But when a tested compound is able to reduce these ions into cuprous or ferrous ions, the indicator rapidly forms a complex with them which is thereafter measured spectrophotometrically.

Metal chelation experiments were performed in 96-well microplates, at least in duplicate, at room temperature. A Synergy HT Multi-Detection Microplate Reader (BioTec Instruments, Inc., USA) was used for these measurements. Detailed methodology was described in our original papers [27, 28]. Shortly, consider the following.

#### 2.2.1. Ferrozine Method

Ferrozine is a specific indicator which forms a magenta coloured complex with ferrous ions; the methodology can be extended for the assessment of total iron chelation after reduction of ferric ions by a suitable reductant like hydroxylamine.

For this assessment of iron chelation, various concentrations of isoflavone (the added concentration was depending on solubility, maximal used concentration was 5 mM, Figure 1 and Table 1) DMSO solutions in acetate (pH 4.5, 5.5) or HEPES (pH 6.8, 7.5) buffer were mixed with ferrous or ferric ions (final concentration of both was 25 *μ*M) for 2 min. After that ferrozine was added in the case of ferrous ions. Hydroxylamine was added prior to ferrozine at pH 7.5 to inhibit ferrous oxidation at this pH. Hydroxylamine solution was used also in the case of total iron chelation at pH 4.5 to reduce remaining ferric ions to ferrous ones which form purple complex with ferrozine afterwards. The absorbance was measured immediately after the addition of ferrozine and 5 min later at 562 nm.

For the determination of the degree of ferric ions reduction, various concentrations of the tested compounds were mixed for 2 min with ferric ions in acetate or HEPES buffer. Afterwards, ferrozine was added and absorbance was measured at 562 nm immediately and 5 min later. Hydroxylamine was used as a positive control (100% reduction).

#### 2.2.2. Hematoxylin Method

Hematoxylin is forming a complex with cupric ions which is thereafter measured spectrophotometrically.

Different concentrations of a tested compound were mixed with cupric ions for 2 min in the presence of a buffer. The mixture was incubated for next 3 min with the indicator hematoxylin in order to enable the reaction of nonchelated copper ions with the indicator. The absorbance was measured thereafter and after another 4 min. Different wavelengths were used according to pH: 595 nm (pH 5.5), 590 nm (pH 6.8), and 610 nm (pH 7.5), as reported in our mentioned paper.

#### 2.2.3. BCS Method

BCS method is analogous to ferrozine method with the exception that BCS is specific for cuprous ions.

Different concentrations of a tested compound (the added concentration was depending on solubility, in few cases 10 mM were used) were mixed with cupric or cuprous ions (the final concentration was 50 *μ*M) and incubated for 2 min in a buffer. In the case of cupric ions, hydroxylamine was added after mixing to reduce nonchelated cupric ions. In the case of cuprous ions, hydroxylamine was added before the copper solution in order to retain copper in its reduced state. The nonchelated copper was then evidenced in both cases by the indicator BCS and the absorbance was read immediately and after 5 min at 484 nm.

The modified BCS method was used for determination of cupric ions reducing potential. Cupric ions were mixed with a tested substance in a buffer without hydroxylamine for 2 min. The reduced copper ions were evidenced by BCS afterwards. Hydroxylamine was used as a positive control (100% copper reduction).

### 2.3. Assessment of Iron/Copper Complex Stoichiometry

The assessment of the stoichiometry followed the previously established protocol [29]. Shortly, all experiments were performed in ultraviolet-transparent cuvettes (BrandTech Scientific Inc., The United Kingdom) and absorbance was measured by the use of spectrophotometer Helios Gamma equipped with VISIONlite software 2.2 (ThermoFisher Scientific Inc., USA). Standard Job's method [30] and the same transition metal solutions were used as mentioned above. In all measurement of cuprous ions at all pH and ferrous ions at pH 7.5, hydroxylamine was added to buffer to retain metal in its reduced state.

A methanolic solution of a tested substance was mixed with a solution of metal ions for 1 min at different molar concentration ratios ranging generally from 1 : 4 to 4 : 1 (substance : metal) at all tested pH conditions. Afterwards absorption spectra were immediately measured. The blank was composed of a buffer and a solvent at the ratio 2 : 1, respectively.

### 2.4. Statistical Analysis

The amount of nonchelated or reduced copper/iron was calculated from the difference of absorbance between the tested sample (with the indicator) and its corresponding blank (without indicator) divided by the difference of the control sample (the known amount of copper without the tested substance) and its control blank. In reduction experiments, hydroxylamine was always used as a positive control (100% reduction).

Data are expressed as mean ± SD. The differences of isoflavones chelation potencies for both copper and iron were checked by 95% confidence (prediction) intervals of chelation curves. Differences between copper reductions caused by isoflavones were assessed by 95% confidence intervals of the linear regression lines. For all statistical approaches GraphPad Prism version 6 for Windows (GraphPad Software, USA) was used.

## 3. Results

**Figure 2:**
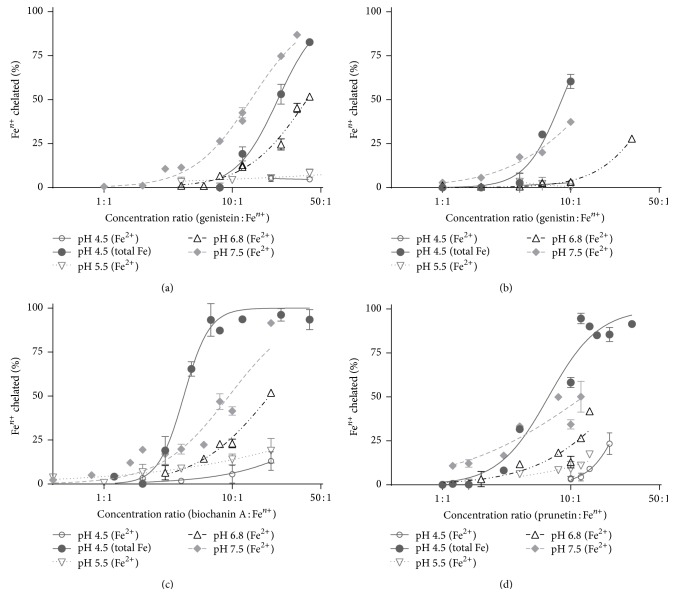
Iron chelation by isoflavonoids. (a) Genistein, (b) genistin, (c) biochanin A, and (d) prunetin.

Previously, we have shown that isoflavonoid genistein is able to chelate iron [31]. Thus we examined other structurally related isoflavones on their iron-chelating capacity. As expected, all isoflavonoids containing isolated hydroxyl or methoxyl groups did not chelate iron at all. Only isoflavones containing the 5-hydroxy-4-keto chelation site (genistein, biochanin A, prunetin, and genistin) were able to chelate iron, although their iron-chelating potency was moderate. They almost did not chelate iron at the ratio of 1 : 1, but their chelation activity at the ratio of 10 : 1 (isoflavone : iron) was with the exception of genistein more than 50% (Figure 2). On the other hand, at the acidic conditions, none of the tested isoflavones was able to substantially chelate ferrous ions. The differences between isoflavones were clearly pronounced for total iron at pH 4.5, where biochanin A was the most active compound followed by prunetin and genistin, and interestingly, genistein was the less efficient compound at these conditions (Figure S1 in Supplementary Material available online at http://dx.doi.org/10.1155/2015/437381). At pH 7.5 and 6.8 the differences were minimal, but biochanin A and prunetin appeared to be again slightly more active than genistein and genistin. At lower pH, the chelation was low and thus no differences were found.

Both of the most common aglycones genistein and daidzein did not reduce iron in our previous experiments [12]. In this study, all other isoflavones were tested using the same setting and, in general, they did not reduce iron as well. The reduction at the highest tested ratio of 20 : 1 was always below 10% (data not shown).

**Figure 3:**
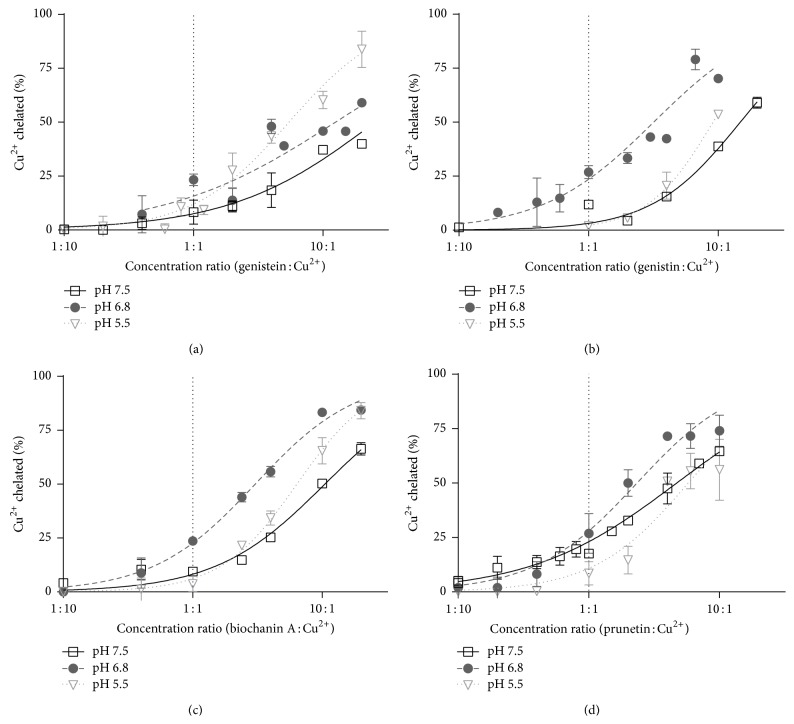
Cupric chelation by active isoflavones measured by the hematoxylin method. (a) Genistein, (b) genistin, (c) biochanin A, and (d) prunetin.

Since the iron-chelating properties of isoflavones were moderate, thus clearly less pronounced than in the case of powerful iron chelators like deferoxamine [27], we firstly tested their cupric chelating properties in a mild competitive environment of hematoxylin using our already published assay [28]. As previously, only isoflavones containing the 5-hydroxy-4-keto chelation site were able to chelate copper (Figure 3). Their copper-chelating properties were similarly to iron only moderate and thus again markedly less expressed than in the case of clinically used copper chelator trientine [28]. At neither pH condition (5.5–7.5) they were able to chelate more than 25% of copper at the ratio of 1 : 1. However, their chelation potencies ranged from 25 to 75% in relation to pH and a tested compound at the ratio of 10 : 1. The detailed analysis using confidence intervals (Figure S2) showed that, at pH 7.5, again biochanin A and particularly prunetin were more active than genistein and genistin. Similarly, genistein appeared to be the least active copper chelator at pH 6.8. On the other hand, the differences were minimal at pH 5.5.

In order to analyse the ability of isoflavones to chelate copper in the more competitive ambient, the BCS method was used. None of the tested isoflavones were able to chelate more than 10% of cupric or cuprous ions at the ratio of 10 : 1 (isoflavone : copper) at any tested pH (4.5–7.5, data not shown).

To analyse the relationship of copper and isoflavones completely, we tested copper reduction at pH 4.5–7.5. In contrast to iron reduction, all isoflavones with the exception of biochanin A and ononin were able to reduce copper. In contrast to low iron reduction, copper reduction was taking place spontaneously in the solvent, particularly in the cases of higher pH.

**Figure 4:**
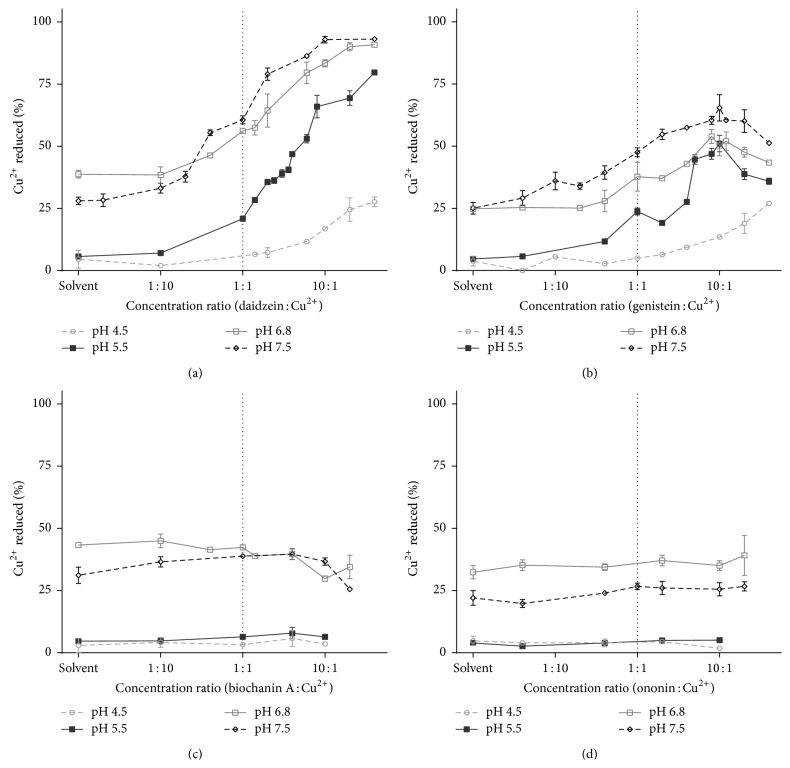
Four possible Cu reduction reactions of isoflavones. (a) Progressive copper reduction, (b) peaked copper reduction, (c) decreased copper reduction, and (d) no interaction.

There were 4 observed reactions of isoflavones (see Figure 4 for examples):

- Progressive copper reduction (Figure 4(a)). This was the most common reaction. The reducing properties of isoflavones were concentration dependent; that is, they increased with the increasing concentration of a tested compound.
- Peaked copper reduction. This case was found in genistein (Figure 4(b)) and prunetin. Both compounds firstly dose-dependently reduced copper, but at some breakpoint ratio, their copper-reducing property decreased with a further increase in isoflavone concentration. This breakpoint ratio was about 10 : 1 in the case of genistein and slightly less in the case of prunetin.
- Decreased copper reduction. This feature was found only in the case of biochanin A (Figure 4(c)). Biochanin A was able to decrease the spontaneous copper reduction at pH 6.8 and 7.5.
- No interaction. In this case, copper reduction was statistically similar to the solvent at all tested concentrations. This reaction was observed only in the case of ononin (Figure 4(d)).

**Figure 5:**
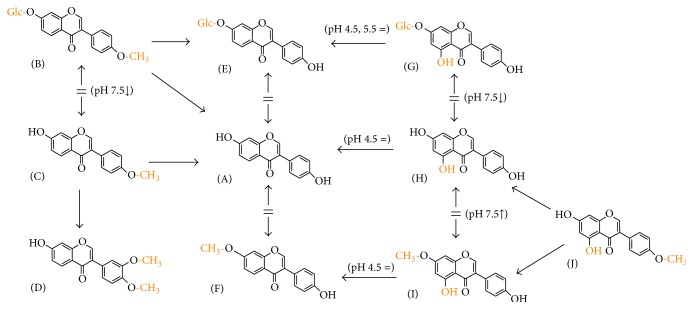
Scheme showing the dependence of structural modification of isoflavone structure on copper reduction at all tested pH. Arrows show the direction of the more potent compound in the sense of copper reduction. Two-way arrows mean no difference. The exceptions are shown in parenthesis. Daidzein (A) was selected as a reference compound because other structural modifications can be related to it. (B) Ononin, (C) formononetin, (D) cladrin, (E) daidzin, (F) isoformononetin, (G) genistin, (H) genistein, (I) prunetin, and (J) biochanin A.

**Figure 6:**
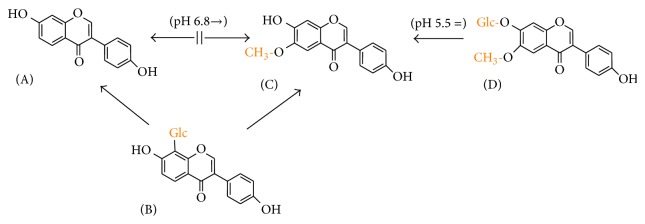
Scheme showing the dependence of structural modification of ring A of isoflavones on copper reduction at all tested pH. Arrows show the direction of the more potent compound in the sense of copper reduction. Two-way arrows mean no difference. The exceptions are shown in parenthesis. (A) Daidzein, (B) puerarin, (C) glycitein, and (D) glycitin.

In order to compare copper-reducing properties, we subtracted the reduction percentages caused by isoflavones from the spontaneous reduction (in the cases of genistein and prunetin, the data after breakpoint ratio were ignored by virtue of facilitating the analysis). Interestingly, we found linear relationship between the ratio of isoflavone to copper and the reduction potential in all cases. This enabled an easy comparison by use of 95% confidence intervals of linear regression curves at all tested pH conditions (examples are shown in Figure S3). Based on these results, we prepared an overview of copper-reducing properties (Figures 5 and 6).

In summary, it appeared that

- the presence of a free 4′-hydroxyl group in ring B was the most important factor for copper reduction; in all cases and at all pH conditions its substitution by a methoxyl decreased the reduction potential (Figure 5). Two adjacent methoxyl groups in ring B had slightly higher reduction potential in comparison to an isolated methoxyl group (cladrin versus formononetin);
- the presence of a free 7-hydroxyl group in ring A had generally no or little copper-reducing potential with exception of pH 7.5 where it apparently increased the reducing potential, for example, ononin versus formononetin or genistein versus genistin/prunetin. The copresence of the 6-methoxyl and 7-hydroxyl group in ring A may slightly increase the reduction potential under some conditions as can be seen at pH 6.8 (daidzein versus glycitein, Figure 6);
- the presence of free 5-hydroxyl group was not associated with copper reduction; on the contrary, it was associated with copper chelation and therefore decreased copper reduction (biochanin A). Its influence was minimal at pH 4.5, where cuprous chelation was low or not present;
- sugars decreased the reduction potential by blocking free hydroxyl groups (e.g., glycitein versus glycitin) or by the steric hindrance (e.g., puerarin versus daidzein).

In order to confirm iron and copper chelation experiments in a competitive ambient, we performed the noncompetitive chelation experiments with genistein, as a chelating representative of isoflavones, by the use of standard Job's method. Ferric chelation experiments showed both at acidic conditions (pH 4.5) and at neutral conditions (7.5) the complex of genistein with ferric ions at the stoichiometry of 2 : 1 (Figure S4). Ferrous chelation experiments demonstrated the same complex at pH 7.5 but no complex was formed at 4.5. Thus we performed additional experiments at pH 5.5 and 6.8; a complex formation was observed but the assessment of stoichiometry was not possible likely due to low affinity of genistein for ferrous ions at these pH conditions. Experiments with cuprous ions in the presence of hydroxylamine in order to block oxidation of cuprous ions revealed that genistein was not able to chelate cuprous ions at any of the tested pH (4.5, 5.5, 6.8, and 7.5, Figure S5). Interestingly, concerning cupric ions at pH 7.5, we were not able to establish the stoichiometry but the complex was clearly formed. At pH 4.5, again no chelation was observed. However, at pH 5.5 and 6.8, the complex of the stoichiometry of 1 : 1 between genistein and cupric ions was clearly seen (Figure S6).

## 4. Discussion

Iron-chelating properties of flavonoids have been tested several times by different research groups and there is a general agreement now that possible chelation sites include two proximal hydroxyl groups (*o*-dihydroxyl group in ring B or ring A), the 3-hydroxy-4-keto or the 5-hydroxy-4-keto position [31–33]. This is apparently valid for isoflavonoids and for copper chelation as well [31, 34]. On the other side, there are few available papers reporting that genistein and biochanin A, both possessing the 5-hydroxy-4-keto group, do not form complexes with copper/iron [13, 14, 35] or daidzein, which does not have any of the mentioned sites, binds copper [15]. All these papers have used a direct spectrophotometric assessment and thus the findings likely have arisen from the fact that (1) the spectra of isoflavonoids are only modestly changed after the addition of copper or iron, (2) the change of spectra caused by “hidden” absorption of cupric or ferric salts is interpreted as a shift in maximal absorption of an isoflavonoid and thus as a complex formation [10, 36], and, moreover, (3) metal chelation by isoflavonoids is relatively low, as can be seen in the current work. This paper has recognized difficulties in the direct noncompetitive spectrophotometric approach as well, because we were not able to ascertain the stoichiometry of Cu^2+^ and genistein complex formation at pH 7.5, notwithstanding repeated measurements. Such obstacles could be traced in the literature data as well; indeed, only one article has reported stoichiometry for genistein and biochanin A with cupric and ferric ions, according to our knowledge. The stoichiometry of the genistein/biochanin A complexes with ferric ions was 2 : 1 (isoflavone : Fe^3+^) at pH 4, 6, and 7.3 in accordance with our data. Such a stoichiometry has been already observed for cupric ions at pH 7.3 while, at pH 4 and 6, the complex of biochanin A or genistein with cupric ions has had the stoichiometry of 1 : 1 [10]. Again, such results are in full accordance with our results.

The applied competitive methodologies as the main approach in this study are based on the competition of a tested isoflavonoid with known ferrous (ferrozine), cupric (hematoxylin), or cuprous (BCS) chelating agents which serve as well as indicators because their complexes with Fe/Cu have distinct absorption. Such methodologies consider better the affinity of a tested compound for metal due to the competition with the indicator. Therefore, they are superior proof of metal chelation potential of a compound than the simple spectrophotometric approach in a noncompetitive ambient, where only a metal ion is present with a tested substance. The results from the current study confirmed that isoflavonoids are forming complexes with both metals but the chelation potential is much lower in comparison to efficient flavonoids like baicalein or standard iron/copper chelators deferoxamine and trientine [31, 34]. In fact, even in the study where no spectral change after addition of Cu^2+^ to genistein solution was found, low copper chelation effect by the use of the hematoxylin method was observed [14]. A novel bit of information is that isoflavones were not able to chelate cuprous ions. This was shown in the present study in both noncompetitive experiments and it has ensued from comparison of hematoxylin and BCS experiments as well since the former formed complexes with cupric ions and the latter only with cuprous ones.

Published studies seem to support our metal reduction results when considering the differences in methodological approach, in particular concentrations of the flavonoid, the metal, and pH [11, 35, 37–39]. A recent study stays at the first sight in a clear contrast to our study, since Ullah et al. have showed copper reduction by genistein and biochanin A, too [38]. However, when analysing the available results from that study in detail, biochanin A was likely not responsible for copper reduction. In that article, stable concentrations of biochanin A and BCS were used and the concentration of copper was changing. In high copper to biochanin A ratios (8 : 1 to 3 : 1) the copper reduction measured as absorbance at 480 nm was apparently the same. However, this reflected rather the spontaneous copper reduction at pH 7.5 (e.g., Figure 4) which was mainly dependent on the stable concentration of BCS. At lower ratios of copper to biochanin A, the absorbance gradually dropped. This may be due to copper-chelating effect of biochanin A but the role of decreasing copper content cannot be neglected. In contrast, we fully agree that genistein reduced copper since the reduction curve of genistein showed clearly higher absorbance than that of biochanin A. But, consistent with our results, it appeared that genistein might decrease the copper reduction in high genistein to copper ratios because the absorbance curve was not linear (linear curve would suggest the Lambert-Beer law between added copper and absorbance). This may indicate bell-shaped behavior observed in this study too (Figure 4(b)).

The impact of pH was also apparent from this study. The influence of pH is opposite when comparing copper and iron. In the case of copper, increasing pH from acidic to neutral raises the reduction potential while, in the case of iron, decreasing pH increases reduction potential. It should be mentioned that solubility of ferric ions is important factor because ferric ions are rapidly transformed into insoluble hydroxide and ferric oxide at neutral pH [40]. Practically, iron is not reduced by isoflavonoids at pH 4.5–5.5 as was reported by us and in another study [35], while at pH 3.6 iron reduction by both iron-chelating and nonchelating isoflavonoids was achieved [11]. The degree of copper reduction was always higher than that of iron, which was in line with known lower standard reduction potential for Cu^2+^/Cu^+^ when compared to that for Fe^3+^/Fe^2+^ couple. Such results were confirmed previously for both flavonoids and isoflavonoids [35]. Another factor which should be considered in particular in the case of very active reducing agents is the time of incubation. Longer incubation means higher reduction and in the cases of highly active reducing agents (in our case daidzein and glycitein), the complete copper reduction (100%) might be achieved. We were aware of this fact and therefore we used 5-minute interval which was sufficient for characterization of isoflavonoids according to their reduction potential.

In agreement with previous studies, the reduction potential was not equal to direct antioxidant activity [11]. This was true for metal chelators because their reduction potential reflected the balance between metal chelation and intrinsic reduction potential which was equivalent to scavenging of free radicals. But when we analyzed isoflavones which did not chelate copper/iron, the relationship between direct scavenging potential and copper reduction seemed to be direct. For both effects, the presence of an unsubstituted free 4′-hydroxyl was the most important factor while a free 7-hydroxyl was partly important only in specific cases [13].

In contrast to data on transition metal chelation and reduction, which are generally in harmony, as can be seen above, information on effect of isoflavonoids on oxidation initiated by transition metals was highly divergent [10–13, 41]. Here, apparently more factors beyond transition metal chelation and reduction played the role. The experimental model seemed to be the major issue. Interestingly, rather prooxidative effect of metal chelating isoflavones and their complexes may be responsible for some of their potentially positive effects. In particular, there were several studies showing that antiproliferative effects of isoflavonoids were boosted by free metals or their complexes with metals, and, in particular, copper reduction was responsible for them [36–38, 42–44]. This could be true for tumor cells, which have generally higher amount of copper [45]. Normal cells were thus contrarily and advantageously less sensitive or insensitive to antiproliferative effect of isoflavones [37, 43]. In general, low oral bioavailability of nonmetabolized isoflavones represents the main drawback of possible flavonoid use for many medicinal purposes [46], but it is not a real limitation in cancer therapy.

Another clinically interesting aspect may represent influence of isoflavones on copper and iron absorption from the gastrointestinal tract. Data on this issue for isoflavonoids are currently not available. Assuming that (1) copper is present in the Cu^2+^ form at physiological pH and in the presence of oxygen and (2) copper is transported in the reduced form, for example,* via* hCTR1, copper has to be reduced in the intestine before the absorption. Dietary components or the action of metalloreductases could be responsible for that [20]. Moreover, it has been shown that vitamin C improved copper absorption [18]. The possible mechanism may be linked to copper reduction. But vitamin C is known to reduce iron as well. Therefore several isoflavones, which are able to reduce copper without any significant influence on iron, could be considered as useful tools in the experiments aimed at understanding the way of copper absorption. This issue requires complex* in vitro/ex vivo* and* in vivo* approaches to really confirm or reject it and it will be analyzed in our next study.

## 5. Conclusions

This study confirmed that isoflavones possessing the 5-hydroxy-4-keto chelation site were able to chelate ferric, ferrous, and cupric ions but they did not chelate cuprous ions. However, their affinity for all mentioned ions was generally lower than those of other active flavonoids or known copper/iron chelators. None of isoflavones was able to reduce substantially ferric ions but all of them with exception of biochanin A and ononin were able to reduce cupric ions. The most important factor for cupric reduction by isoflavones was the presence of a free 4′-hydroxyl group; contrarily the presence of a free 5-hydroxyl group decreased or abolished the reduction due to chelation of cupric ions.

## Supplementary Material

Supplementary materials include comparison of iron and copper chelating activity of tested isoflavones by use of 95% prediction bands (S1,2), comparison of isoflavones by means of confidence intervals of linear regression lines of the relationship between copper reduction and the ratio isoflavone to copper at pH 7.5 (S3), and assessment of genistein-ferric and genistein-cupric ions complex stoichiometry by the use of Job's method (S4, 5, 6).

## Figures and Tables

**Table 1:** 

Isoflavonoids	R_5_	R_6_	R_7_	R_8_	R_3′_	R_4′_
Biochanin A	OH	H	OH	H	H	O-CH_3_
Cladrin	H	H	OH	H	O-CH_3_	O-CH_3_
Daidzein	H	H	OH	H	H	OH
Daidzin	H	H	O-Glc	H	H	OH
Formononetin	H	H	OH	H	H	O-CH_3_
Genistein	OH	H	OH	H	H	OH
Genistin	OH	H	O-Glc	H	H	OH
Glycitein	H	O-CH_3_	OH	H	H	OH
Glycitin	H	O-CH_3_	O-Glc	H	H	OH
Isoformononetin	H	H	O-CH_3_	H	H	OH
Ononin	H	H	O-Glc	H	H	O-CH_3_
Prunetin	OH	H	O-CH_3_	H	H	OH
Puerarin	H	H	OH	Glc	H	OH

Glc: glucose.

## Appendix

See Supplementary Material.

## Abbreviations

BCS: Bathocuproinedisulfonic acid disodium salt
HEPES: 4-(2-Hydroxyethyl)-1-piperazineethanesulfonic acid.
