# Supplementary material for: Isoflavones Reduce Copper with Minimal Impact on Iron In Vitro
Source: Oxid Med Cell Longev. 2015 Jul 26;2015:437381. doi: 10.1155/2015/437381 (PMC4529972; doi:10.1155/2015/437381)
Supplement: Supplementary file 1 — Supplementary materials include comparison of iron and copper chelating activity of tested isoflavones by use of 95% prediction bands (S1,2), comparison of isoflavones by means of confidence intervals of linear regression lines of the relationship between copper reduction and the ratio isoflavone to copper at pH 7.5 (S3), and assessment of genistein-ferric and genistein-cupric ions complex stoichiometry by the use of Job's method (S4, 5, 6). [file 437381.f1.pdf]

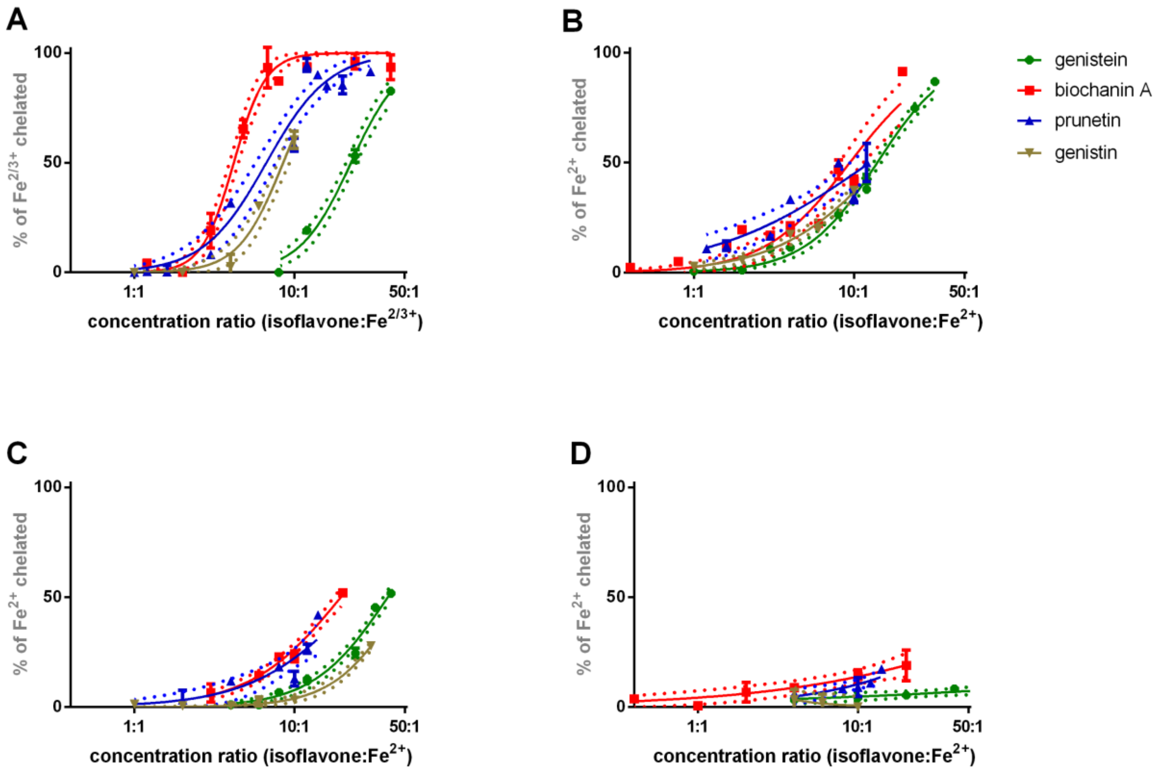

FIGURE S1. Comparison of iron-chelating activity of the tested isoflavones by use of 95% prediction bands. **A**: total iron chelation at pH 4.5, **B**: ferrous chelation at pH 7.5, **C**: ferrous chelation at pH 6.8 and **D**: ferrous chelation at pH 5.5.

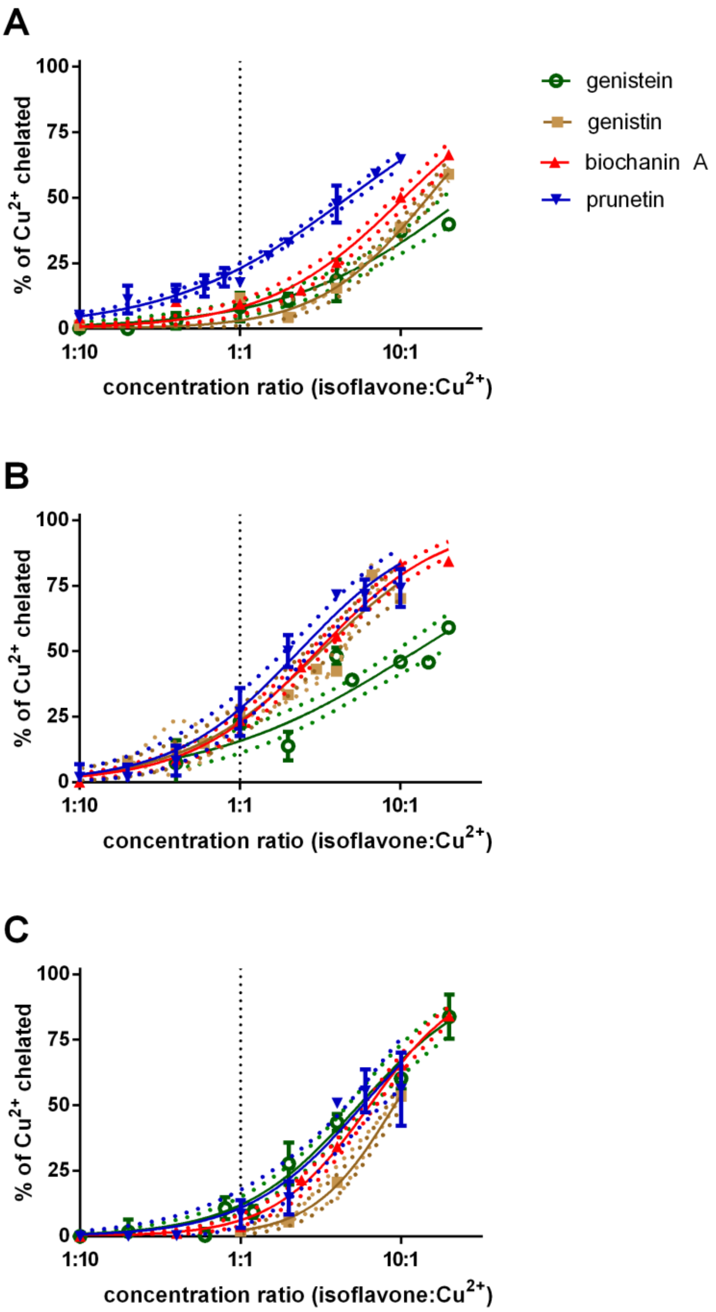

FIGURE S2. Comparison of cupric-chelating activity of tested isoflavones by use of 95% prediction bands (the hematoxylin method). **A**: pH 7.5, **B**: pH 6.8 and **C**: pH 5.5.

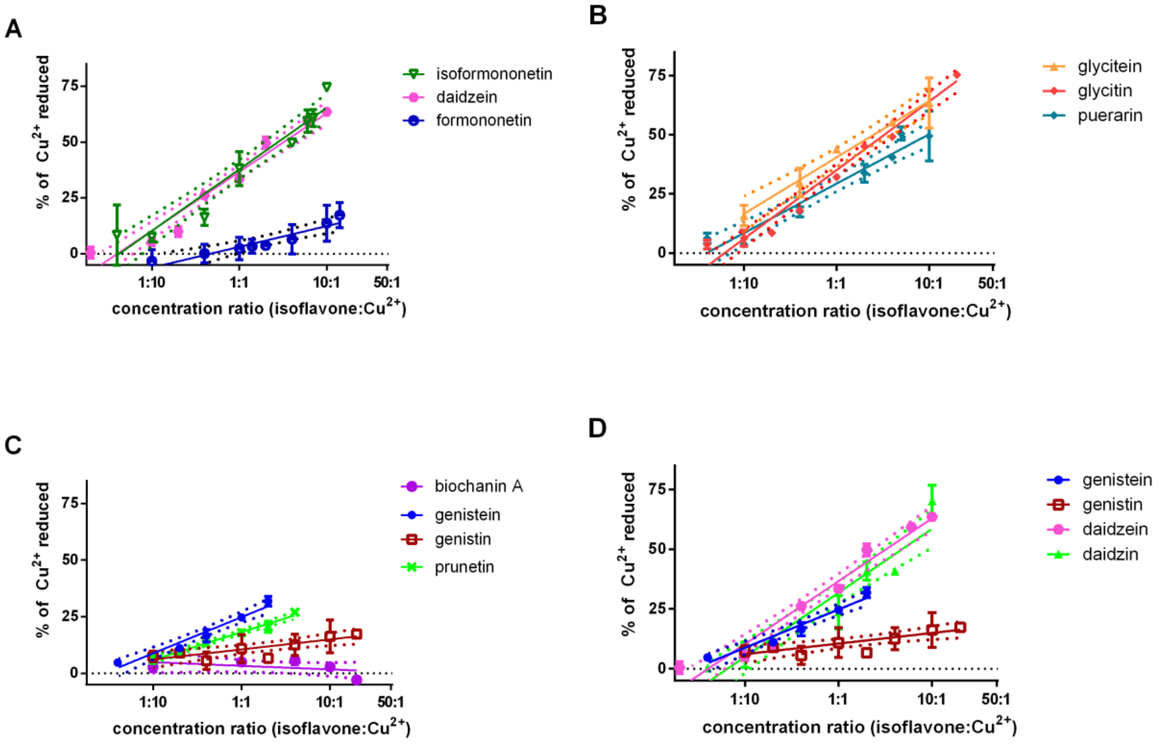

FIGURE S3. Comparison of isoflavones by means of confidence intervals of linear regression lines of the relationship between copper reduction and the ratio isoflavone to copper at pH 7.5. **A-D**: comparisons of different compounds.

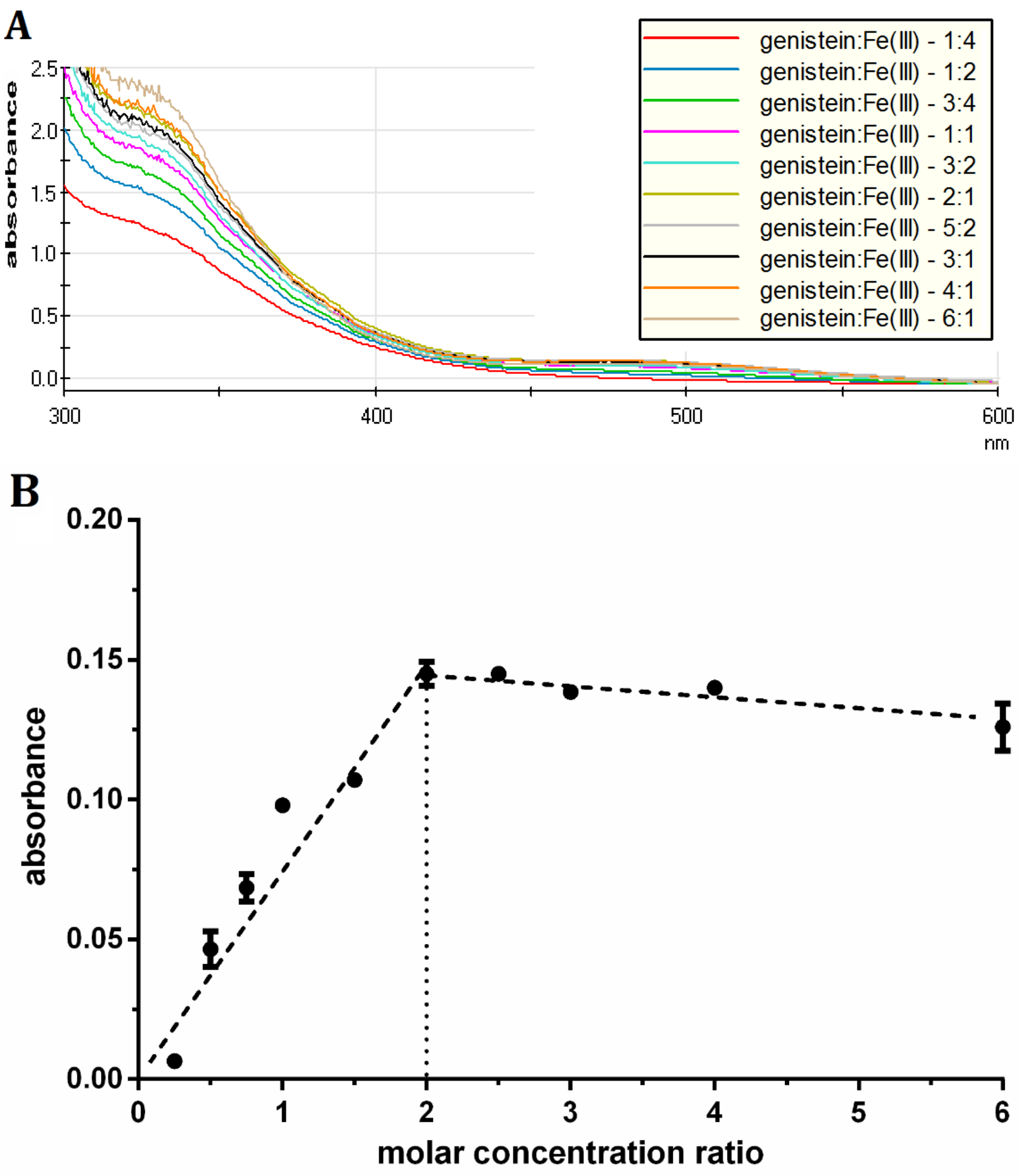

FIGURE S4. Assessment of genistein-ferric ions complex stoichiometry by the use of Job's method. **A**: measured spectra at pH 7.5, **B**: Job's plot.

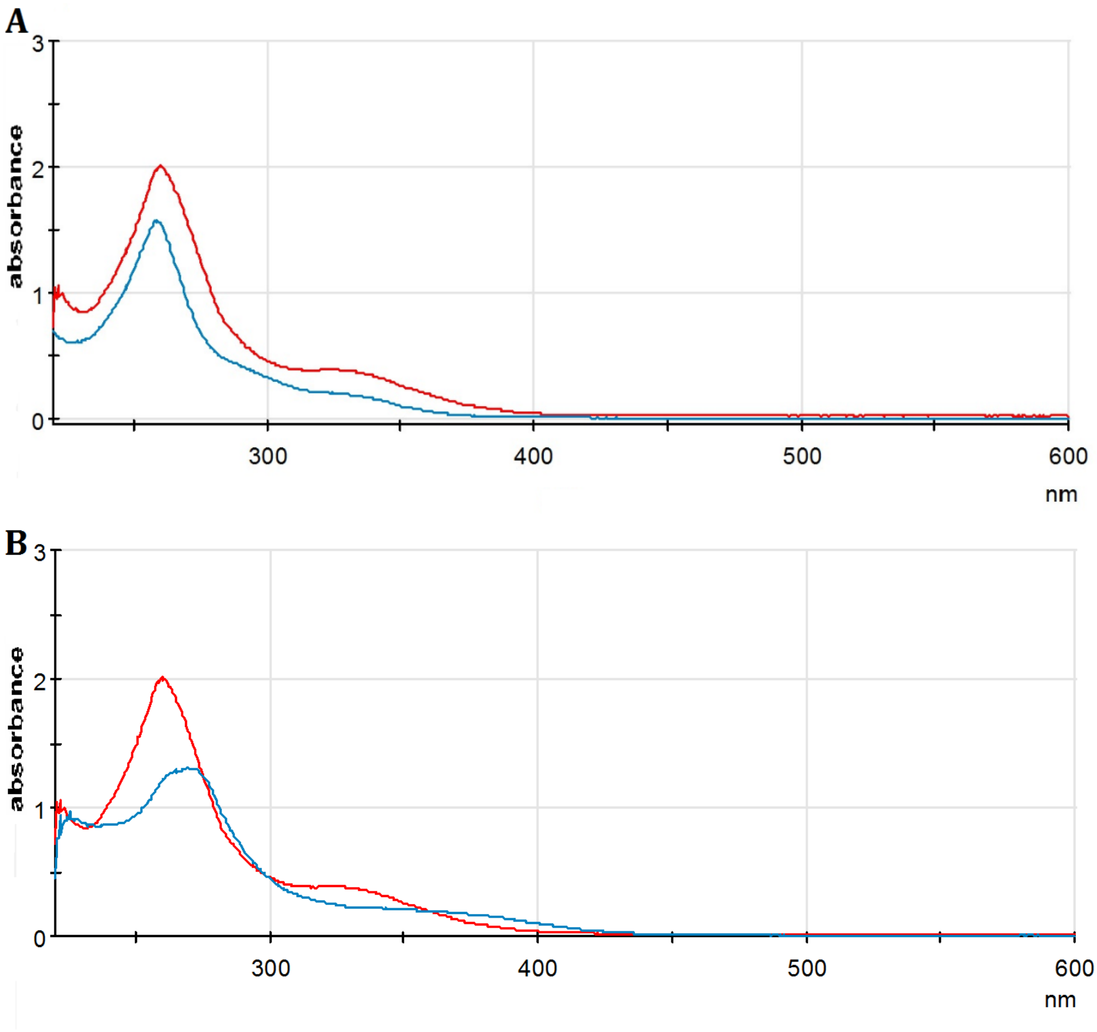

FIGURE S5. Spectra of genistein alone and after addition of cuprous (**A**) and cupric ions (**B**) at pH 7.5. Genistein in a concentration of 40 μM is shown in red, its spectra after addition of copper ions in blue (genistein was in a concentration of 33 μM with copper in an excessive concentration of 500 μM). After addition of cuprous ions, no complex was formed (**A**) in contrast to addition of cupric ions (**B**), where the spectrum was clearly changed suggesting complex formation.

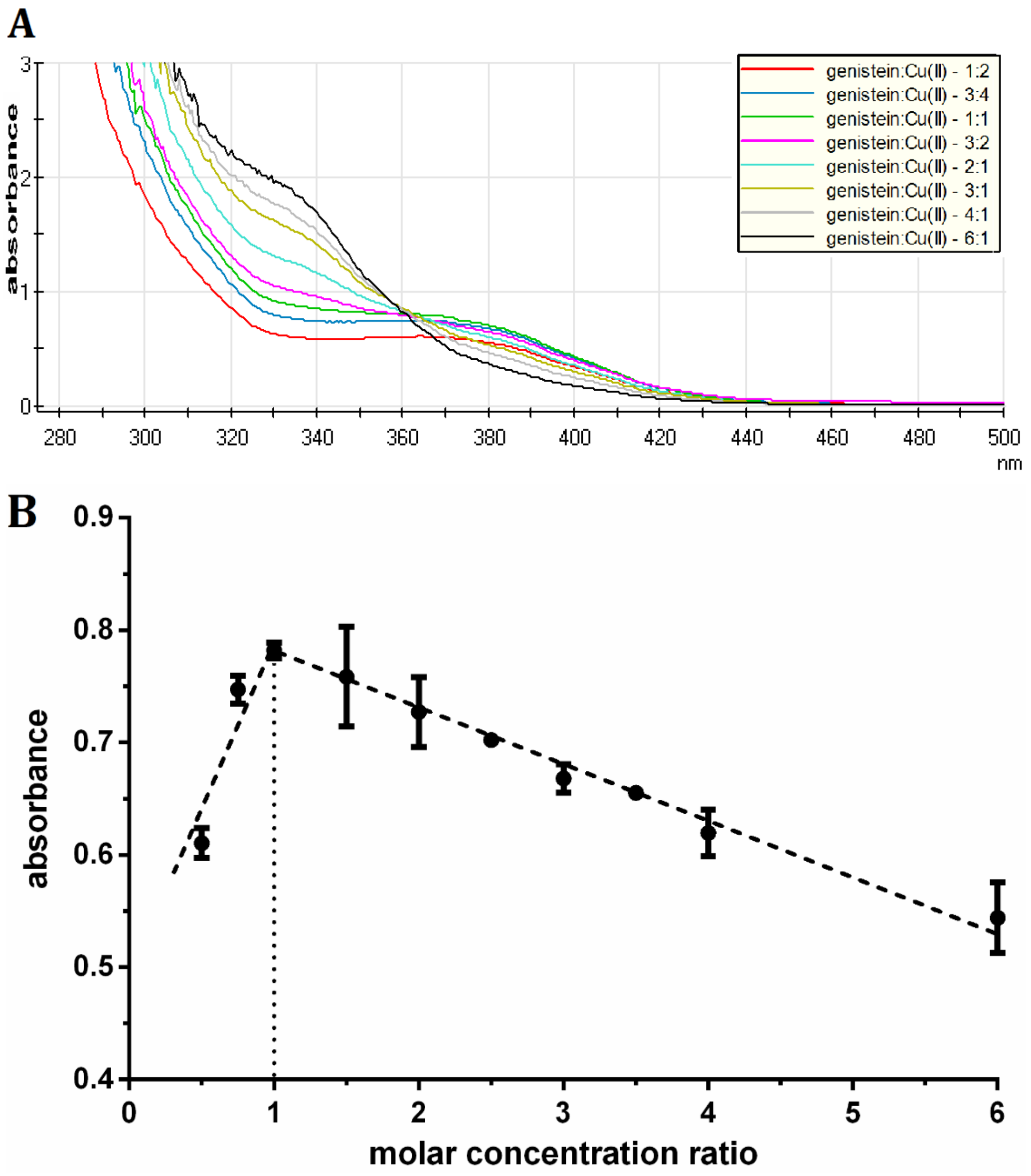

FIGURE S6. Assessment of genistein-cupric ions complex stoichiometry by Job's method. **A**: measured spectra at pH 6.8, **B**: Job's plot.
